# Supplementary material for: Structural and Spectroscopic Study of New Copper(II) and Zinc(II) Complexes of Coumarin Oxyacetate Ligands and Determination of Their Antimicrobial Activity
Source: Molecules. 2023 Jun 5;28(11):4560. doi: 10.3390/molecules28114560 (PMC10254212; doi:10.3390/molecules28114560)
Supplement: Supplementary file 1 [file molecules-28-04560-s001.zip › molecules-2384191-supplementary.pdf]

Supplementary Materials

Structural and Spectroscopic Study of New Copper(II) and Zinc(II) Complexes of Coumarin Oxyacetate Ligands and Determination of their Antimicrobial Activity

Muhammad Mujahid [1], Natasha Trendafilova [2], Georgina Rosair [3], Kevin Kavanagh [4], Maureen Walsh [1], Bernadette S. Creaven [1,5,*] and Ivelina Georgieva [2,*]

[1] Centre of Applied Science for Health, TU Dublin, Tallaght, Dublin D24 FKT9, Ireland
[2] Institute of General and Inorganic Chemistry, Bulgarian Academy of Sciences, 11 Acad. G. Bonchev Str., Sofia, Bulgaria
[3] School of Engineering & Physical Sciences, Heriot-Watt University, Edinburgh, Scotland, UK EH14 4AS
[4] Department of Biology, Maynooth University, Co. Kildare, Ireland
[5] School of Chemical and Pharmaceutical Sciences, TU Dublin, Central Quad, GrangeGorman Dublin D07 H6K8, and Centre of Applied Science for Health, TU Dublin, Tallaght, Dublin D24 FKT9, Ireland

Table S1. Substituents, their positions, atom number assignment for ^{1}H and ^{13}C NMR spectra and abbreviations for 2-(2-oxo-2*H*-chromen-substituted-yl)oxy acetic acids.

IUPAC Name of the compound/Abbreviation (Number)	-R³	-R⁴	-R⁵	-R⁶	-R⁷	-R⁸
2-[(2-Oxo-2*H*-chromen-3-yl)oxy]acetic acid/C-3oxy-acetH **(1)**	OCH_2COOH	H	H	H	H	H
2-[(2-Oxo-2*H*-chromen-4-yl)oxy]acetic acid/C-4oxy-acetH **(2)**	H	OCH_2COOH	H	H	H	H
2-[(6-Chloro-2-oxo-2*H*-chromen-4-yl)oxy]acetic acid/6Cl-C-4oxy-acetH **(3)**	H	OCH_2COOH	H	Cl	H	H
2-[(4-Methyl-2-oxo-2*H*-chromen-6-yl)oxy]acetic acid/4Me-C-6oxy-acetH **(4)**	H	CH_3^*	H	OCH_2COOH	H	H
2-[(2-Oxo-2*H*-chromen-7-yl)oxy]acetic acid/C-7oxy-acetH **(5)**	H	H	H	H	OCH_2COOH	H
2-[(4-Methyl-2-oxo-2*H*-chromen-7-yl)oxy]acetic acid/4Me-C-7oxy-acetH **(6)**	H	CH_3^*	H	H	OCH_2COOH	H
2-[(3,4,8-Trimethyl-2-oxo-2*H*-chromen-7-yl)oxy]acetic acid/3,4,8-triMe-C-7oxy-acetH **(7)**	CH_3^*	CH_3''	H	H	OCH_2COOH	CH_3'''
2-[(3-Chloro-4-methyl-2-oxo-2*H*-chromen-7-yl)oxy]acetic acid/3Cl-4Me-C-7oxy-acetH **(8)**	Cl	CH_3^*	H	H	OCH_2COOH	H
2-{[4-(Trifluoromethyl)-2-oxo-2*H*-chromen-7-yl]oxy}acetic acid/4CF₃-C-7oxy-acetH **(9)**	H	CF_3^*	H	H	OCH_2COOH	H
2-[(8-Acetyl-2-oxo-2*H*-chromen-7-yl)oxy]acetic acid/8acetyl-C-7oxy-acetH **(10)**	H	H	H	H	OCH_2COOH	CH_3CO

-OCH_2COOH = 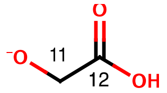

-CH_3CO = 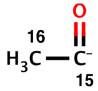

CF_3^*/CH_3^* = Assigned 15 to carbon and its protons.

CH_3'' = Assigned 16 to carbon and its protons.

CH_3''' = Assigned 17 to carbon and its protons.

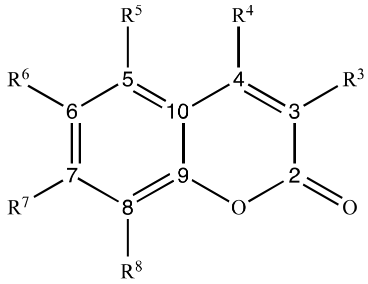

Atoms and substituents position for coumarin moiety.

Table S2. Molar conductivity of Zn(II) and Cu(II) complexes along with corresponding ligands recorded in DMSO.

Ligand	ΛM (Scm^2mol^{-1}) 25°C / 37°C	Zinc(II) complex	ΛM (Scm^2mol^{-1}) 25°C / 37°C	Cu(II) complex	ΛM (Scm^2mol^{-1}) 25°C / 37°C
C-3oxy-acetH **(1)**	0.11 / 0.27	Zn(C-3oxy-acet)$_2$(H_2O)$_4$ **(11)**	1.13 / 1.45	Cu(C-3oxy-acet)$_2$(H_2O)$_4$ **(21)**	1.77 / 2.11
C-4oxy-acetH **(2)**	0.11 / 0.20	Zn(C-4oxy-acet)$_2$(H_2O)$_4$ **(12)**	1.19 / 1.47	Cu(C-4oxy-acet)$_2$(H_2O)$_4$ **(22)**	2.17 / 2.33
6Cl-C-4oxy-acetH **(3)**	0.13 / 0.23	Zn(6Cl-C-4oxy-acet)$_2$(H_2O)$_4$ **(13)**	1.29 / 1.52	Cu(6Cl-C-4oxy-acet)$_2$(H_2O)$_4$ **(23)**	2.38 / 2.62
4Me-C-6oxy-acetH **(4)**	0.10 / 0.19	Zn(4Me-C-6oxy-acet)$_2$(H_2O)$_4$ **(14)**	1.17 / 1.41	[Cu(4Me-C-6oxy-acet)$_2$(H_2O)$_4$] **(24)**	1.88 / 2.05
C-7oxy-acetH **(5)**	0.12 / 0.26	Zn(C-7oxy-acet)$_2$(H_2O)$_4$ **(15)**	1.23 / 1.38	Cu(C-7oxy-acet)$_2$(H_2O)$_4$ **(25)**	1.67 / 1.93
4Me-C-7oxy-acetH **(6)**	0.10 / 0.22	Zn(4Me-C-7oxy-acet)$_2$(H_2O)$_4$ **(16)**	1.11 / 1.35	Cu(4Me-C-7oxy-acet)$_2$(H_2O)$_4$ **(26)**	1.96 / 2.17
3,4,8triMe-C-7oxy-acetH **(7)**	0.10 / 0.18	Zn(3,4,8triMe-C-7oxy-acet)$_2$(H_2O)$_4$ **(17)**	1.15 / 1.33	[Cu(3,4,8-triMe-C-7oxy-acet)$_2$(H_2O)$_4$] **(27)**	2.07 / 2.72
3Cl-4Me-C-7oxy-acetH **(8)**	0.11 / 0.23	Zn(3Cl-4Me-C-7oxy-acet)$_2$(H_2O)$_4$ **(18)**	1.28 / 1.45	Cu(3Cl-4Me-C-7oxy-acet)$_2$(H_2O)$_4$ **(28)**	2.97 / 3.08
4CF_3-C-7oxy-acetH **(9)**	0.10 / 0.20	Zn(4CF_3-C-7oxy-acet)$_2$(H_2O)$_4$ **(19)**	1.39 / 1.53	Cu(4CF_3-C-7oxy-acet)$_2$(H_2O)$_4$ **(29)**	1.42 / 1.68
8acetyl-C-7oxy-acetH **(10)**	0.10 / 0.23	Zn(8acetyl-C-7oxy-acet)$_2$(H_2O)$_4$ **(20)**	1.22 / 1.39	Cu(8acetyl-C-7oxy-acet)$_2$(H_2O)$_4$ **(30)**	1.21 / 1.55

Table S3. Crystal structure data and structure refinement details of [Zn(C-4oxy-acet)$_2$(H_2O)$_4$] **(12).**

Empirical formula	$C_{22}H_{22}ZnO_{14}$
Formula weight	575.77 g/mol
Temperature	100(2) K
Wavelength	0.71073 Å
Crystal system	Triclinic
Space group	*P*1
Unit cell dimensions	*a* = 4.8200 (4) Å, *b* = 10.0615 (9) Å, *c* = 12.0670 (11) Å
	α = 96.052 (6)°, β = 96.580 (5)°, γ = 94.819 (5)°
Volume	575.37 (9) Å^3
Z	**1**
Density	1.662 mg m^{-3}
Absorption coefficient	1.14 mm^{-1}
Crystal size	0.58 × 0.24 × 0.20 mm^3
Index range	*h* = -6→7
	k = -14→15
	l = -18→18
Theta range of data collection	2.5-33.5°
Reflections collected	15014
Independent reflections	4501
Absorption correction	multi-scan *SADABS* (Bruker, 2009)
Refinement method	Least-squares full matrix on F^2
Data/ restrains/ parameters	4501/ 0/ 181
Goodness -of-fit on F^2	1.028
Largest difference in peak and hole	0.47 and -0.89 e Å^{-3}

Table S4. Crystal data and structural refinement for [Zn(4CF$_3$-C-7oxy acet)$_2$(H$_2$O)$_4$].2H$_2$O **(19).**

Empirical formula	$C_{24}H_{24}F_6O_{16}Zn$
Formula weight	747.83 g/mol
Temperature	100(2) K
Wavelength	0.71073 Å
Crystal system	Triclinic
Space group	$P\bar{1}$
Unit cell dimensions	*[1]*
Volume	744.45(10) Å^3
Z	1
Density	1.454 mg m^{-3}
Absorption coefficient	0.916 mm^{-1}
Crystal size	0.90 × 0.10 × 0.04 mm^3
Index range	h = -7→7<br>k = -9→9<br>l = -36→33
Theta range of data collection	1.67-65.28°
Reflections collected	16776
Independent reflections	5317[R(int) = 0.0499]
Absorption correction	*SADABS*-2008/1 (Bruker,2008)
Data/ restrains/ parameters	5317/ 9/ 264
Goodness -of-fit on F^2	0.933
Final R indices [I>2sigma(I)]	R1 = 0.0517, wR2 = 0.1272
R indices (all data)	R1 = 0.0821, wR2 = 0.1505
Largest difference in peak and hole	0.826 and -1.464 e Å^{-3}

Table S5. Hydrogen bond parameters for [Zn(C-4oxy-acet)$_2$(H_2O)$_4$] **(12)** and [Zn(4CF_3-C-7oxy-acet)$_2$(H_2O)$_4$].2H_2O (**19**).

[Zn(C-4oxy-acet)$_2$(H_2O)$_4$][a] **(12)**				
D-H···*A*	*D*-H (Å)	H···*A* (Å)	*D*···*A* (Å)	*D*-H···*A* (°)
O1-H11*W*···O4^{i}	0.88 (2)	1.850 (2)	2.6831 (14)	158.3 (18)
O1-H12*W*···O2^{ii}	0.88 (2)	2.070 (2)	2.8627 (14)	150.1 (17)
O1-H12*W*···O3^{ii}	0.88 (2)	2.765 (19)	3.2809 (14)	118.8 (15)
O2-H21*W*···O6^{iii}	0.85 (2)	1.940 (2)	2.7538 (14)	161.1 (18)
O2-H21*W*···O7^{iii}	0.85 (2)	2.700 (2)	3.4361 (14)	145.2 (16)
O1-H12*W*···O3^{ii}	0.88 (2)	2.765 (19)	3.2809 (14)	118.8 (15)
[Zn(4CF_3-C-7oxy-acet)$_2$(H_2O)$_4$].2H_2O[b] (**19**)				
D-H···*A*	*D*-H (Å)	H···*A* (Å)	*D*···*A* (Å)	*D*-H···*A* (°)
O1-H1A···O1^{i}	0.846(10)	2.129(16)	2.927(5)	157(3)
O1-H1B···O3^{ii}	0.855(10)	1.900(18)	2.690(3)	153(3)
O1*W*-H1*W*B···O3*W*^{iii}	0.830(5)	2.370(5)	3.142(3)	156(4)
O1*W*-H1*W*A···O3*W*	1.010(7)	1.790(7)	2.791(3)	170(5)
O3*W*-H3*W*A···O11^{iv}	0.790(4)	2.440(4)	3.196(3)	160(4)
O2A-H2AA···O2^{v}	0.870(10)	1.924(14)	2.788(9)	172(4)

[a] Symmetry transformations used to generate equivalent atoms: (i) -*x*+1, -*y*, -*z*; (ii) -*x*, -*y*, -*z*; (iii) *x*+1, *y*, *z*+1

[b] Symmetry transformations used to generate equivalent atoms:(i) 3-*x*, 3-*y*, 2-*z*; (ii) 3-*x*, 2-*y*, 2-*z*; (iii) 1+*x*, +*y*, +*z*; (iv) +*x*, -1+*y*, +*z*; (v) 2-*x*, 2-*y*, 2-*z*.

Table S6. Selected experimental and calculated bond lengths (in Å) of $[Zn(C\text{-}4oxy\text{-}acet)_2(H_2O)_4]$ **(12)** complex.

Bond	Experiment[this work]	Calc (solid state)[a]	Calc (gas phase)[b]
$C\text{-}C_{carb}$	1.516	1.511	1.527
$C\text{-}O_{carb}$	1.290	1.295	1.277
$C{=}O_{carb}$	1.236	1.253	1.255
ΔR(OCO)	0.054	0.042	0.022
$C{=}O_{coum}$	1.230	1.245	1.230
$Zn\text{-}O_{coum}$	2.073	2.093	2.044

[a] periodic calculations with PAW-PBE method using VASP code.

[b] calculations at DFT/B3LYP/C**B1** level of theory (see Computational Details).

Table S7. Room temperature magnetic moment values of copper(II) complexes of 2-(2-oxo-2*H*-chromen-substituted-yl)oxy acetic acids.

Complex	μ_{eff} B.M./Cu
[Cu(C-3oxy-acet)$_2$.4H$_2$O] **(21)**	1.77
[Cu(C-4oxy-acet)$_2$.4H$_2$O] **(22)**	1.83
[Cu(6Cl-C-4oxy-acet)$_2$.4H$_2$O] **(23)**	1.91
[Cu(4Me-C-6oxy-acet)$_2$.4H$_2$O] **(24)**	1.72
[Cu(C-7oxy-acet)$_2$.4H$_2$O] **(25)**	1.79
[Cu(4Me-C-7oxy-acet)$_2$.4H$_2$O] **(26)**	1.80
[Cu(3,4,8-triMe-C-7oxy-acet)$_2$.4H$_2$O] **(27)**	1.85
[Cu(3Cl-4Me-C-7oxy-acet)$_2$.4H$_2$O] **(28)**	1.88
[Cu(4CF$_3$-C-7oxy-acet)$_2$.4H$_2$O] **(29)**	1.92
[Cu(8acetyl-C-7oxy-acet)$_2$.4H$_2$O] **(30)**	1.82

Table S8. MIC_{50} values (in μM) for Zn(II) and Cu(II) complexes.

Compound	*C. albicans* (μM±SEM)	*P. aeruginosa* (μM±SEM)	*MRSA* (μM±SEM)
Zinc(II)complexes (**11-20**)	Inactive	Inactive	Inactive
Copper(II)complexes (**21-30**)	Inactive	Inactive	Inactive
Copper(II) acetate monohydrate	Inactive	Inactive	Inactive
Zinc(II) acetate	Inactive	Inactive	Inactive
Dimethyl sulfoxide (DMSO)	Inactive	Inactive	Inactive
Vancomycin		66.28±2.4	1.17±0.09
Amphotericin B	4.26±0.35		

(a)

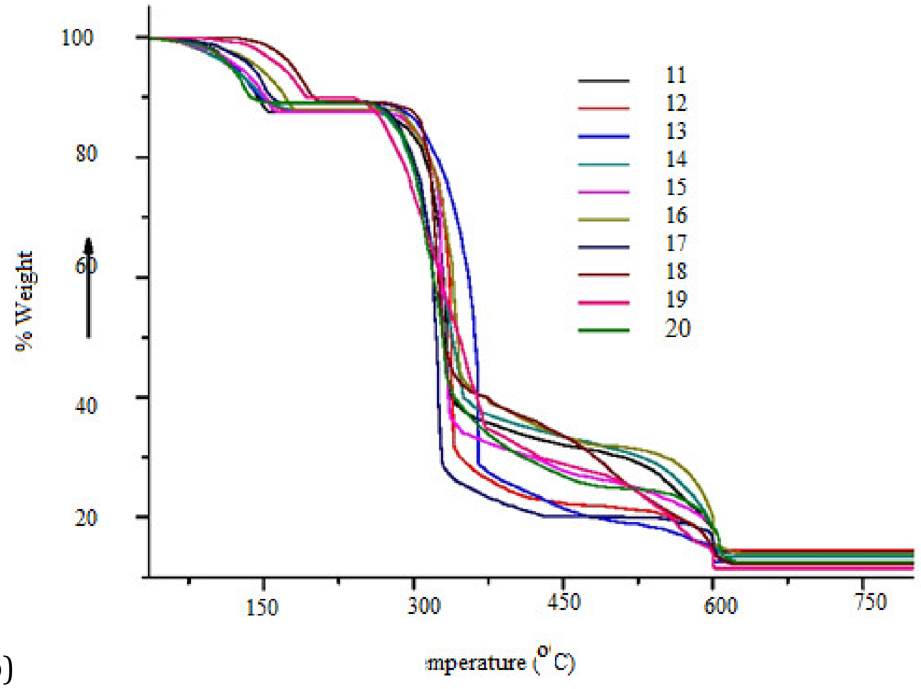

(b)

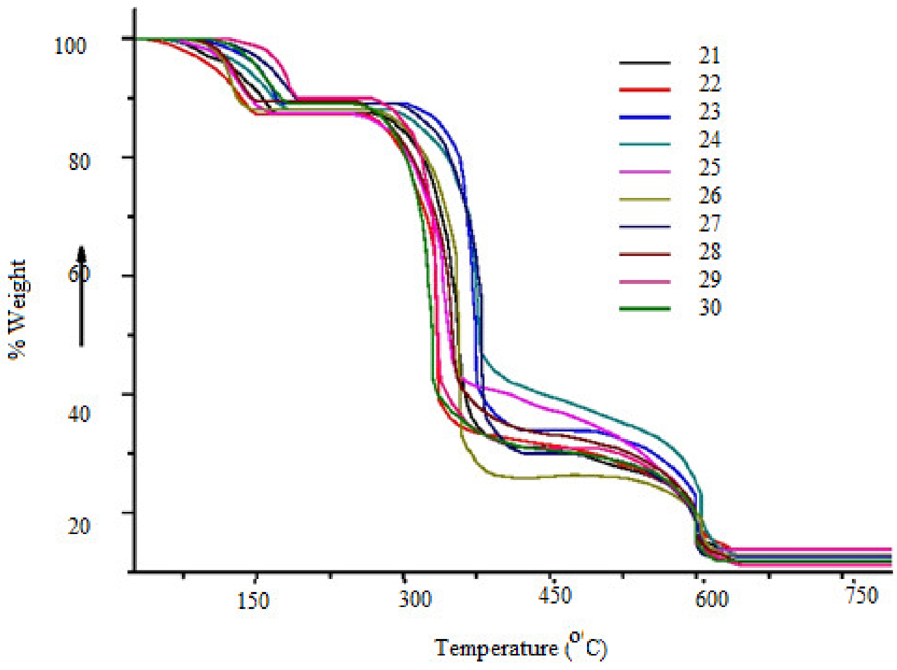

Figure S1. Thermogravimetric curves of (a) zinc(II) complexes (**11-20**) and (b) copper(II) complexes (**21-30**).

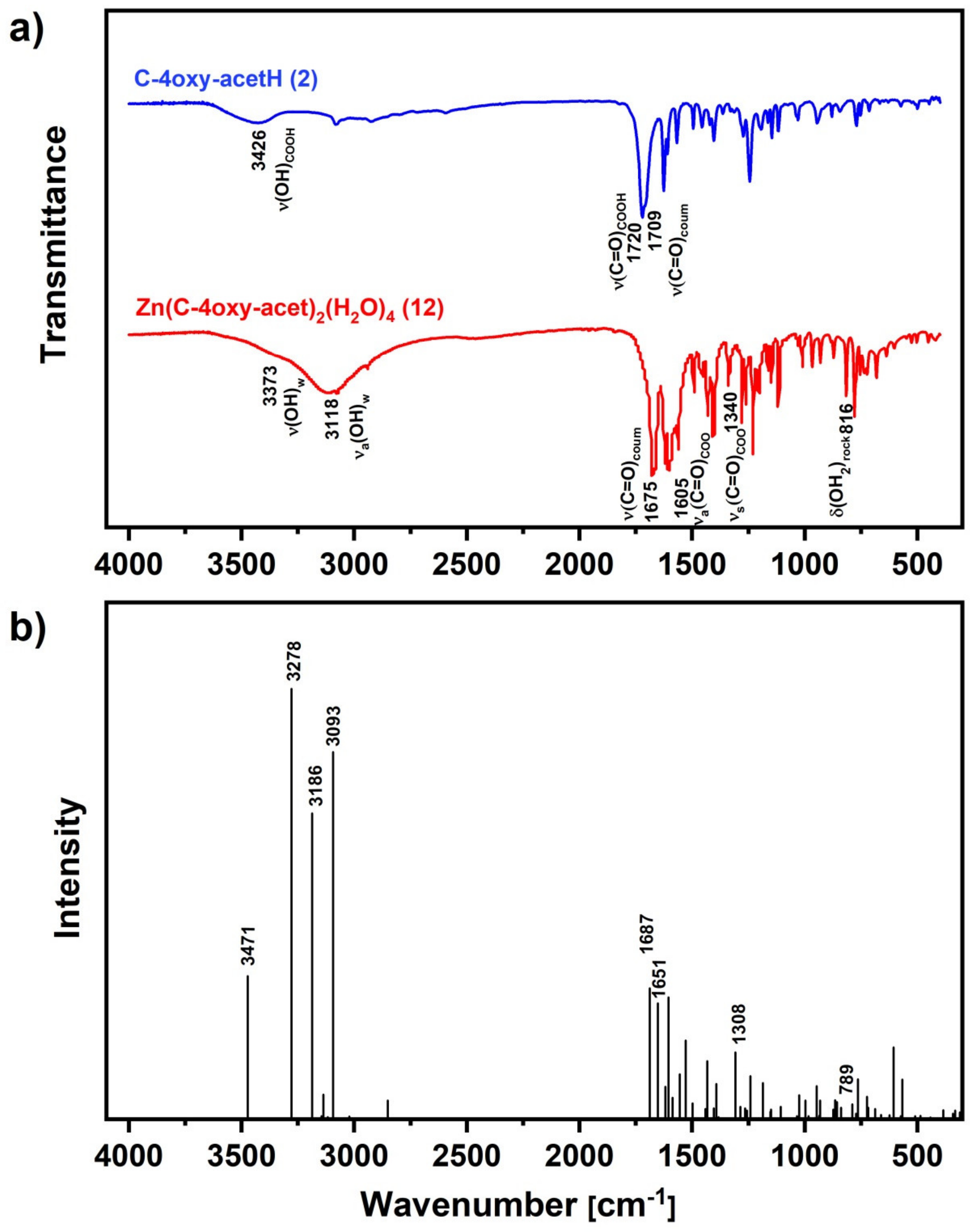

Figure S2. Experimental IR spectra of C-4oxy-acetH (**2**) and its Zn(II) complex (**12**) (a) compared to the calculated IR spectrum for the model Zn(C-4oxy-acet)$_2$(H_2O)$_4$ complex in solid state (b).

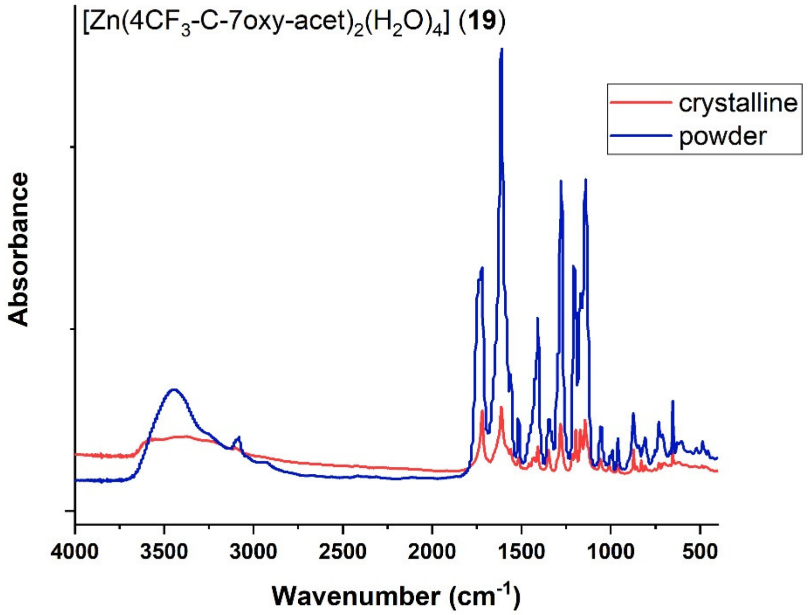

Figure S3. Compared experimental IR spectra of the crystalline (red) and powder (blue) [Zn(4CF$_3$-C-7oxy-acet)$_2$(H$_2$O)$_4$] **(19)** complex.

(a)

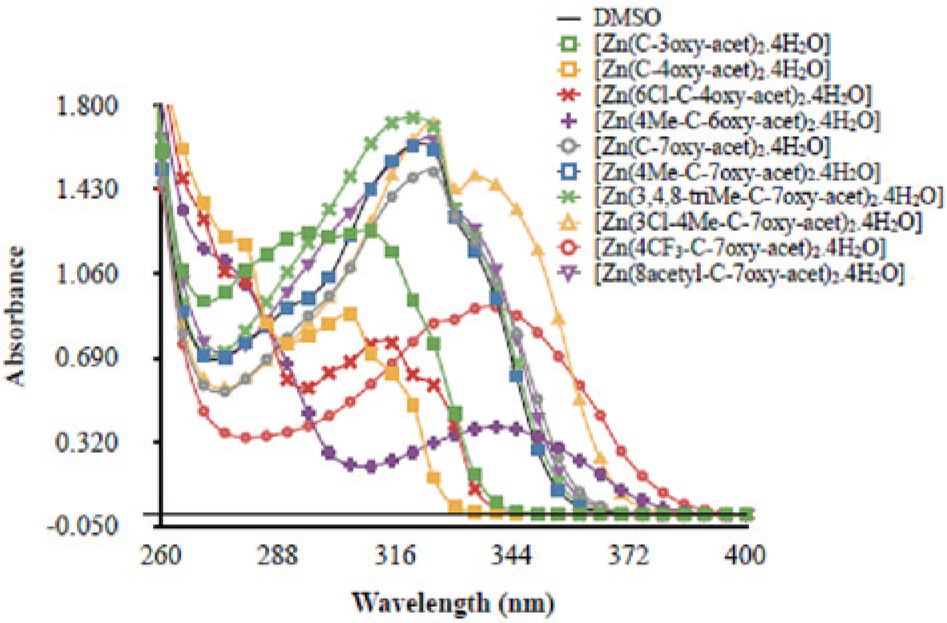

(b)

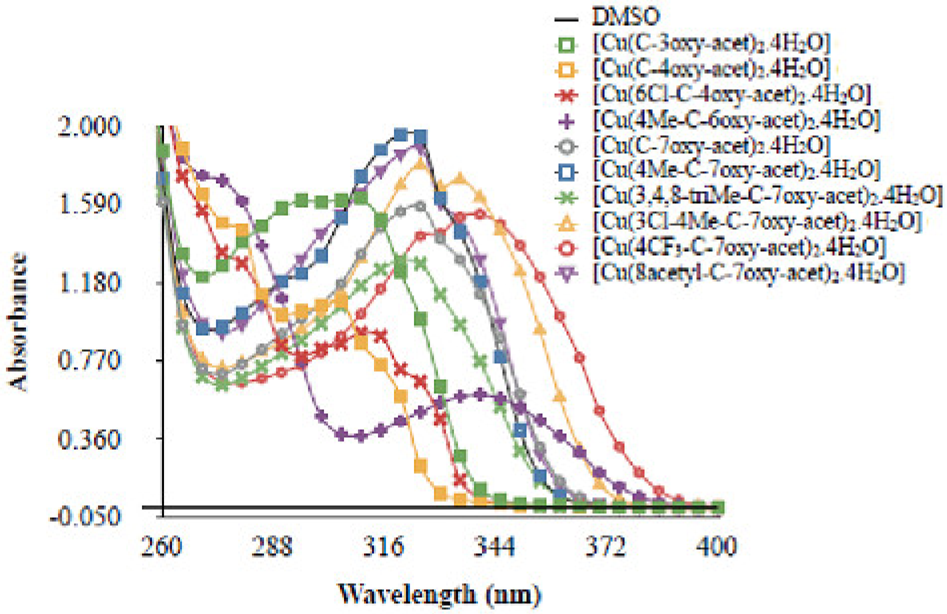

Figure S4. UV-Vis spectra of 2-(2-oxo-2*H*-chromen-substituted-yl)oxy acetic acid based zinc(II) complexes (**11-20**) (a) and copper(II) complexes (**21-30**) (b) recorded over the wavelength range 260 to 400 nm in DMSO at a concentration of 5.0x10^{-5} M.

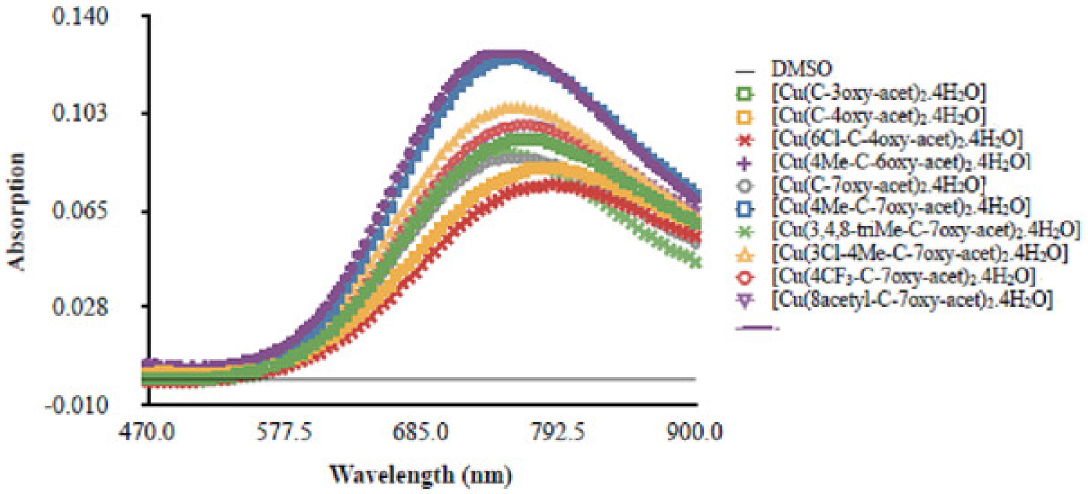

Figure S5. d-d transition band of copper(II) complexes of 2-(2-oxo-2*H*-chromen-substituted-yl)oxy acetic acids (**21**-**30**) recorded over the wavelength range 470 to 900 nm in DMSO at a concentration of $5.0x10^{-5}$ M.

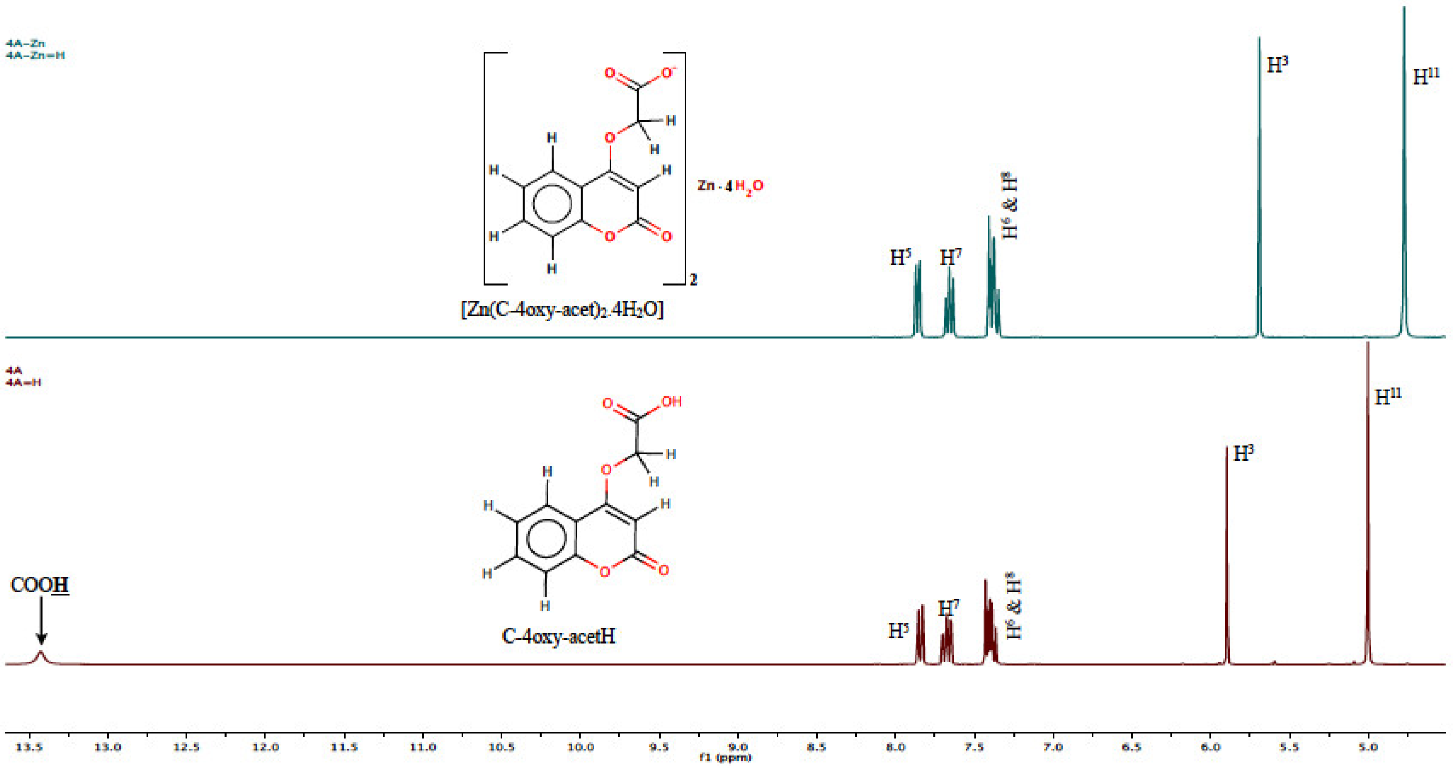

Figure S6. ^{1}H NMR spectra of [Zn(C-4oxy-acet)$_2$(H_2O)$_4$] complex (**12)** along with corresponding ligand **2** recorded in DMSO-*d*$_6$.

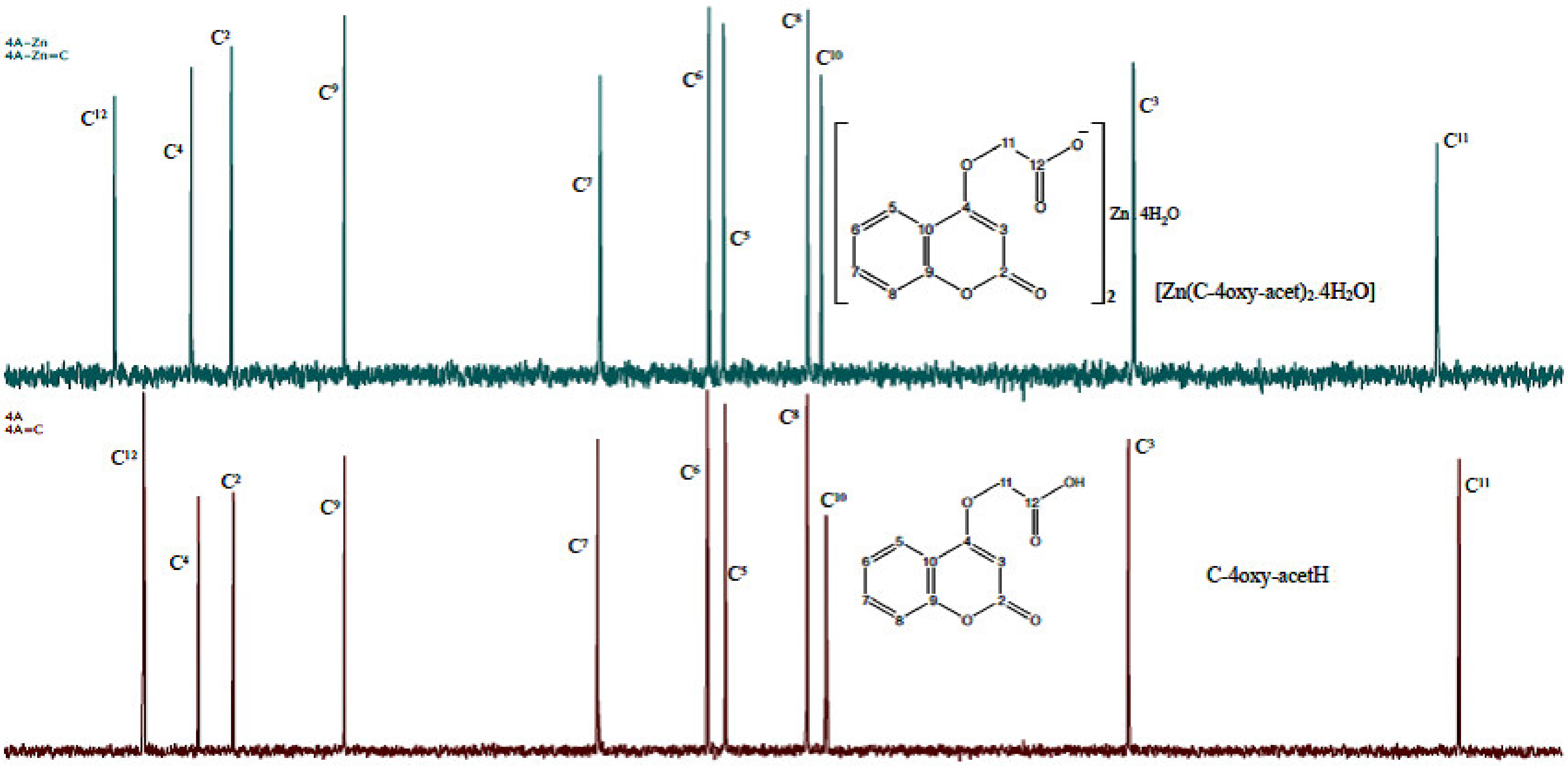

Figure S7. ^{13}C NMR spectra of of [Zn(C-4oxy-acet)$_2$(H_2O)$_4$] complex (**12)** along with corresponding ligand **2** recorded in DMSO-d_6.

Synthesis of 2-(2-oxo-2*H*-chromen-substituted-yl)oxyacetato zinc(II) complexes.

The appropriate 2-(2-oxo-2*H*-chromen-substituted-yl)oxy acetic acid (1.0 mmol) was dissolved in hot absolute ethanol (20 ml). Anhydrous zinc(II) acetate (0.09 g, 0.50 mmol) was dissolved in deionised water (5 ml) at room temperature and mixed immediately with the ligand solution. The resulting mixture was stirred at reflux temperature for 3 h. Occasionally the reflux condenser was removed to release the acetic acid generated during the reaction. Upon cooling, the reaction mixture yielded a white precipitate. This was filtered and washed with deionised water (30 ml x 2), then with absolute ethanol (10 ml x 2) and finally dried in a vacuum oven for 36 h at 40 °C. The experimental data recorded for complexes **11-20** are given in the following paragraphs.

S1.1. bis-{2-(2-Oxo-2H-chromen-3-yl)oxyacetatotetraaqua} zinc(II)

[Zn(C-3oxy-acet)$_2$.4H$_2$O] (**11**)

Ligand = C-3oxy-acetH **(1)** (0.22 g, 1.0 mmol). **Yield** = 0.19 g, 68%. **M.P.** = 275 °C (decomposed). **Solubility** = DMSO only. **CHN:** Calculated for $C_{22}H_{22}ZnO_{14}$ %C, 45.89%, %H, 3.85%, %Zn, 11.36; Found %C, 45.56, %H, 3.28, %Zn, 11.60. δ_H (300 MHz, DMSO-d_6) 7.53 [1H, dd, *J* = 7.7 Hz, 1.4 Hz, H^5], 7.38 [1H, m, H^7], 7.23 [2H, m, H$_{aromatic}$], 7.10 [1H, s, H^4], 4.56 [2H, s, H^{11}]. δ_C (125 MHz, DMSO-d_6) C^{12} not observed, 156.6 [C^2], 148.9 [C^9], 142.8 [C^3], 128.3 [C^7], 126.9 [C^5], 124.6 [C^6], 119.7 [C^{10}], 115.5 [C^8], 114.4 [C^4], 66.9 [C^{11}]. **UV-Vis (DMSO)** λ = 310 nm ε = 24360 M^{-1}cm^{-1}, λ = 295 nm ε = 24400 M^{-1}cm^{-1}. **Λ_M (DMSO)** = 1.13 Scm^{2}mol^{-1}. **FTIR (KBr)** 3517, 3053, 2919, 1730, 1627, 1576, 1458, 1424, 1382, 1266, 1165, 847, 752 cm^{-1}.

S1.2. bis-{2-(2-Oxo-2H-chromen-4-yl)oxyacetatotetraaqua} zinc(II)

*[Zn(C-4oxy-acet)$_2$.4H$_2$O] (**12**)*

Ligand = C-4oxy-acetH **(2)** (0.22 g, 1.0 mmol). **Yield** = 0.18 g, 65%. **M.P.** = 260 °C (decomposed). **Solubility** = DMSO only. **CHN:** Calculated for $C_{22}H_{22}ZnO_{14}$ %C, 45.89%, %H, 3.85%, %Zn, 11.36; Found %C, 45.90, %H, 3.49, %Zn, 11.13. δ_H (300 MHz, DMSO-d_6) 7.86 [1H, dd, *J* = 7.9 Hz, 1.4 Hz, H^5], 7.66 [1H, m, H^7], 7.38 [2H, m, H$_{aromatic}$], 5.69 [1H, s, H^3], 4.77 [1H, s, H^{11}]. δ_C (75 MHz, DMSO-d_6) 170.8 [C^{12}], 164.8 [C^4], 161.7[C^2], 152.8 [C^9], 132.7 [C^7], 124.2 [C^6], 123.1 [C^5], 116.4 [C^8], 115.4 [C^{10}], 90.9 [C^3], 67.1 [C^{11}]. **UV-Vis (DMSO)** λ = 320 nm ε = 9080 M^{-1}cm^{-1}, λ = 305 nm ε = 17040 M^{-1}cm^{-1}, λ = 280 nm ε = 23420 M^{-1}cm^{-1}. **Λ_M (DMSO)** = 1.19 Scm^{2}mol^{-1}. **FTIR (KBr)** 3118, 3075, 2941, 1675, 1621, 1605, 1561, 1453, 1431, 1405, 1278, 1230, 870, 816, 779 cm^{-1}.

S1.3. bis-{2-(6-Chloro-2-oxo-2H-chromen-4-yl)oxyacetatotetraaqua} zinc(II)

*[Zn(6Cl-C-4oxy-acet)$_2$.4H$_2$O] (**13**)*

Ligand = 6Cl-C-4oxy-acetH **(3)** (0.25 g, 1.0 mmol). **Yield** = 0.22 g, 72%. **M.P.** = 270 °C (decomposed).

Solubility = DMSO only, **CHN:** Calculated for $C_{22}H_{20}Cl_2ZnO_{14}$ %C, 40.99%, %H, 3.13%, %Zn, 10.15; Found %C, 40.73, %H, 3.03, %Zn, 10.07. δ_H (300 MHz, DMSO-d_6) 7.80 [1H, d, J = 2.6 Hz, H^5], 7.71 [1H, dd, J =8.8 Hz, 2.6 Hz, H^7], 7.45 [1H, d, J = 8.9 Hz, H^8], 5.78 [1H, s, H^3], 4.76 [2H, s, H^{11}]. δ_C (75 MHz, DMSO-d_6) 170.5 [C^{12}], 163.6 [C^4], 161.2 [C^2], 151.4 [C^9], 132.4 [C^7], 128.3 [C^6], 122.2 [C^5], 118.6 [C^8], 116.9 [C^{10}], 91.8 [C^3], 67.3 [C^{11}]. **UV-Vis (DMSO)** λ = 325 nm ε = 11140 $M^{-1}cm^{-1}$, λ = 315 nm ε = 14540 $M^{-1}cm^{-1}$, λ = 280 nm ε = 20320 $M^{-1}cm^{-1}$. **Λ_M (DMSO)** = 1.29 Scm^2mol^{-1}. **FTIR (KBr)** 3426, 3080, 1718, 1625, 1563, 1436, 1425, 1374, 1292, 1251, 941, 846, 823, 530 cm^{-1}.

S1.4. bis-{2-(4-Methyl-2-oxo-2H-chromen-6-yl)oxyacetatotetraaqua} zinc(II)
*[Zn(4Me-C-6oxy-acet)$_2$. 4H$_2$O] (**14**)*

Ligand = 4Me-C-6oxy-acetH **(4)** (0.23 g, 1.0 mmol). **Yield** = 0.19 g, 64%. **M.P.** = 280 ° (decomposed). **Solubility** = DMSO only. **CHN:** Calculated for $C_{24}H_{26}ZnO_{14}$ %C, 47.47%, %H, 4.34%, %Zn, 10.83; Found %C, 47.75, %H, 4.69, %Zn, 10.73. δ_H (300 MHz, DMSO-d_6) 7.24 [1H, d, J = 9.0 Hz, H^8], 7.13 [1H, dd, J = 9.0 Hz, 2.9 Hz, H^7], 7.06 [1H, d, J = 2.9 Hz, H^5], 6.32 [1H, d, J = 1.2 Hz, H^3], 4.52 [2H, s, H^{11}], 2.36 [3H, d, J = 1.2 Hz, H^{15}]. δ_C (75 MHz, DMSO-d_6) 172.8 [C^{12}], 159.9 [C^2], 154.6 [C^6], 153.0 [C^4], 147.1 [C^9], 119.9 [C^{10}], 119.5 [C^5], 117.2 [C^8], 114.5 [C^3], 108.9 [C^7], 66.6 [C^{11}], 18.1 [C^{15}]. **UV-Vis (DMSO)** λ = 340 nm ε = 7140 $M^{-1}cm^{-1}$, λ = 275 nm ε = 21900 $M^{-1}cm^{-1}$. **Λ_M (DMSO)** = 1.17 Scm^2mol^{-1}. **FTIR (KBr)** 3431, 3080, 2983, 1712, 1624, 1573, 1493, 1428, 1385, 1275, 1175, 1062, 825 cm^{-1}.

S1.5. bis-{2-(2-Oxo-2H-chromen-7-yl)oxyacetatotetraaqua} zinc(II)
*[Zn(C-7oxy-acet)$_2$.4H$_2$O] (**15**)*

Ligand = C-7oxy-acetH **(5)** (0.22 g, 1.0 mmol). **Yield** = 0.18 g, 66%. **M.P.** = 280 °C (decomposed). **Solubility** = DMSO only. **CHN:** Calculated for $C_{22}H_{22}ZnO_{14}$ %C, 45.89%, %H, 3.85%, %Zn, 11.36; Found %C, 45.52, %H, 3.54, %Zn, 11.03. δ_H (300 MHz, DMSO-d_6) 7.96 [1H, d, J = 9.4 Hz, H^4], 7.57 [1H, d, J = 8.6 Hz, H^5], 6.89 [1H, dd, J = 8.6 Hz, 2.5 Hz, H^6], 6.83 [1H, d, J = 2.4 Hz, H^8], 6.26 [1H, d, J = 9.5 Hz, H^3], 4.58 [2H, s, H^{11}]. δ_C (75 MHz, DMSO-d_6) 172.3 [C^{12}], 161.6 [C^7], 160.3 [C^2], 155.2 [C^9], 144.3 [C^4], 129.3 [C^5], 112.8 [C^3], 112.4 [C^{10}], 112.2 [C^6], 101.4 [C^8], 66.5 [C^{11}]. **UV-Vis (DMSO)** λ = 335 nm ε = 23200 $M^{-1}cm^{-1}$, λ = 325 nm ε = 29580 $M^{-1}cm^{-1}$. **Λ_M (DMSO)** = 1.23 Scm^2mol^{-1}. **FTIR (KBr)** 3415, 3050, 1692, 1614, 1560, 1419, 1363, 1274, 1238, 1144, 1057, 846 cm^{-1}.

*S1.6. bis-{2-(4-Methyl-2-oxo-2H-chromen-7-yl)oxyacetatotetraaqua} zinc(II) [Zn(4Me-C-7oxy-acet)$_2$.4H$_2$O] (**16**)*

Ligand = 4Me-C-7oxy-acetH **(6)** (0.23 g, 1.0 mmol). **Yield** = 0.19 g, 68%. **M.P.** = 290 °C (decomposed). **Solubility** = DMSO only. **CHN:** Calculated for $C_{24}H_{26}ZnO_{14}$ %C, 47.74%, %H, 4.34%, %Zn, 10.83; Found %C, 47.53, %H, 4.29, %Zn, 10.44. δ_H (300 MHz, DMSO-*d*$_6$) 7.61 [1H, d, *J* = 8.8 Hz, H^5], 6.90 [1H, dd, *J* = 8.8 Hz, 2.5 Hz, H^6], 6.81 [1H, d, *J* = 2.5 Hz, H^8], 6.17 [1H, d, *J* = 1.2 Hz, H^3], 4.58 [2H, s, H^{11}], 2.36 [3H, d, *J* = 1.1 Hz, H^{15}]. δ_C (75 MHz, DMSO-*d*$_6$) 172.3 [C^{12}], 161.5 [C^2], 160.2 [C^7], 154.5 [C^9], 153.4 [C^4], 126.2 [C^5], 113.0 [C^{10}], 112.5 [C^3], 111.0 [C^6], 101.4 [C^8], 66.5 [C^{11}], 18.1 [C^{15}]. **UV-Vis (DMSO)** λ = 335 nm ε = 22560 M^{-1}cm^{-1}, λ = 320 nm ε = 31900 M^{-1}cm^{-1}. Λ_M **(DMSO)** = 1.11 Scm^{2}mol^{-1}. **FTIR (KBr)** 3431, 3081, 1725, 1614, 1560, 1509, 1424, 1391, 1370, 1288, 1152, 1079, 849 cm^{-1}.

*S1.7. bis-{2-(3,4,8-Trimethyl-2-oxo-2H-chromen-7-yl)oxyacetatotetraaqua} zinc(II) [Zn(3,4,8-triMe-C-7oxy-acet)$_2$.4H$_2$O] (**17**)*

Ligand = 3,4,8-triMe-C-7oxy-acetH **(7)** (0.26 g, 1.0 mmol). **Yield** = 0.20 g, 65%. **M.P.** = 285 °C (decomposed). **Solubility** = DMSO only. **CHN:** Calculated for $C_{28}H_{34}ZnO_{14}$ %C, 50.96, %H, 5.91, %Zn, 9.91; Found %C, 50.62, %H, 5.73, %Zn, 10.78. δ_H (300 MHz, DMSO-*d*$_6$) 7.37 [1H, d, *J* = 8.9 Hz, H^5], 6.76 [1H, d, *J* = 9.0 Hz, H^6], 4.58 [2H, s, H^{11}], 2.27 [3H, s, H^{16}], 2.21 [3H, s, H^{17}], 2.03 [3H, s, H^{15}]. δ_C (75 MHz, DMSO-*d*$_6$) 172.6 [C^{12}], 161.3 [C^2], 158.2 [C^7], 150.3 [C^9], 146.9 [C^4], 122.6 [C^5], 117.3 [C^3], 113.4 [C^{10}], 111.9 [C^8], 108.4 [C^6], 66.8 [C^{11}], 14.8 [C^{16}], 12.9 [C^{15}], 8.2 [C^{17}]. **UV- Vis (DMSO)** λ = 335 nm ε = 24260 M^{-1}cm^{-1}, λ = 320 nm ε = 34340 M^{-1}cm^{-1}. Λ_M **(DMSO)** = 1.15 Scm^{2}mol^{-1}. **FTIR (KBr)** 3444, 2925, 1695, 1608, 1561, 1500, 1458, 1382, 1346, 1286, 817, 1134, 762 cm^{-1}.

*S1.8. bis-{2-(3-Chloro-4-methyl-2-oxo-2H-chromen-7-yl)oxyacetatotetraaqua}zinc(II) [Zn(3Cl-4Me-C-7oxy-acet)$_2$.4H$_2$O] (**18**)*

Ligand = 3Cl-4Me-C-7oxy-acetH **(8)** (0.27 g, 1.0 mmol). **Yield** = 0.22 g, 68%. **M.P.** = 275 °C (decomposed). **Solubility** = DMSO only. **CHN:** Calculated for $C_{24}H_{24}Cl_2ZnO_{14}$ %C, 42.85, %H, 3.6, %Zn, 9.72; Found %C, 42.33, %H, 3.65, %Zn, 9.93. δ_H (300 MHz, DMSO-*d*$_6$) 7.69 [1H, d, *J* = 9.0 Hz, H^5], 6.94 [1H, dd, *J* = 8.9 Hz, 2.5 Hz, H^6], 6.85 [1H, d, *J* = 2.5 Hz, H^8], 4.58 [2H, s, H^{11}], H^{15} not observed. δ_C (75 MHz, DMSO-*d*$_6$) 172.1 [C^{12}], 161.5 [C^7], 156.4 [C^2], 152.4 [C^9], 148.8 [C^4], 126.7 [C^5], 116.0 [C^3], 113.1 [C^{10}], 112.6 [C^6], 101.4 [C^8], 66.6 [C^{11}], 16.1 [C^{15}]. **UV-Vis (DMSO)** λ = 335 nm ε = 29100 M^{-1}cm^{-1}, λ = 325 nm ε = 33680 M^{-1}cm^{-1}. Λ_M **(DMSO)** = 1.28 Scm^{2}mol^{-1}. **FTIR (KBr)** 3437, 3082, 2925, 1734, 1618, 1572, 1508, 1425, 1381, 1291, 1205, 1157, 1082, 1009, 831, 753 cm^{-1}.

*S1.9. bis-{2-(4-(Trifluoromethyl)-2-oxo-2H-chromen-7-yl)oxyacetatotetraaqua} zinc(II) [Zn(4CF$_3$-C-7oxy-acet)$_2$.4H$_2$O] (**19**)*

Ligand = 4CF$_3$-C-7oxy-acetH **(9)** (0.29 g, 1.0 mmol). **Yield** = 0.22 g, 62%. **M.P**. = 300 °C (decomposed). **Solubility** = DMSO only. **CHN:** Calculated for $C_{24}H_{20}F_6ZnO_{14}$ %C, 40.5, %H, 2.83, %Zn, 9.19; Found %C, 40.86, %H, 2.73, %Zn, 9.32. δ_H (300 MHz, DMSO-*d*$_6$) 7.55 [1H, dd, *J* = 8.9 Hz, 1.9 Hz, H^5], 7.00 [1H, dd, *J* = 8.9 Hz, 2.5 Hz, H^6], 6.96 [1H, d, *J* = 2.4 Hz, H^8], 6.80 [1H, s, H^3], 4.62 [2H, s, H^{11}]. δ_C (75 MHz, DMSO-*d*$_6$) 171.9 [C^{12}], 162.3 [C^2], 158.7 [C^7], 155.6 [C^9], 139.5 [q, J^{2F} = 31.9 Hz, C^4], 125.6 [C^5], 121.6 [q, J^{1F} = 275.5 Hz, C^{15}], 113.7 [C^6], 113.0 [q, J^{3F} = 5.5 Hz, C^3], 106.2 [C^{10}], 102.4 [C^8], 66.6 [C^{11}]. **UV-Vis (DMSO)** λ = 340 nm ε = 17760 M^{-1}cm^{-1}. **Λ$_M$ (DMSO)** = 1.39 Scm^{2}mol^{-1}. **FTIR (KBr)** 3448, 3103, 1720, 1613, 1560, 1408, 1385, 1349, 1280, 1196, 1170, 1144, 1059, 872, 842, 807 cm^{-1}.

*S1.10. bis-{2-(8-Acetyl-2-oxo-2H-chromen-7-yl)oxyacetatotetraaqua} zinc(II) [Zn(8acetyl-C-7oxy-acet)$_2$.4H$_2$O] (**20**)*

Ligand = 8acetyl-C-7oxy-acetH **(10)** (0.26 g, 1.0 mmol). **Yield** = 0.19 g, 60%. **M.P**. = 280 °C (decomposed). **Solubility** = DMSO only. **CHN:** Calculated for $C_{26}H_{26}ZnO_{16}$ %C, 47.32, %H, 3.97, %Zn, 9.91; Found %C, 47.06, %H, 3.91, %Zn, 9.97. **δ_H (300 MHz, DMSO-*d*$_6$)** 7.96 [1H, d, *J* = 9.6 Hz, H^4], 7.62 [1H, d, *J* = 8.8 Hz, H^5], 6.96 [1H, d, *J* = 8.8 Hz, H^6], 6.30 [1H, d, *J* = 9.5 Hz, H^3], 4.67 [2H, s, H^{11}], 2.56 [3H, s, H^{16}]. δ_C (75 MHz, DMSO-*d*$_6$) 199.3 [C^{15}], 172.0 [C^{12}], 159.5 [C^2], 157.7 [C^7], 150.2 [C^9], 144.2 [C^4], 129.9 [C^5], 118.5 [C^8], 112.9 [C^3], 112.4 [C^{10}], 109.6 [C^6], 66.8 [C^{11}], 32.1 [C^{16}]. **UV-Vis (DMSO)** λ = 335 nm ε = 24680 M^{-1}cm^{-1}, λ = 325 nm ε = 32440 M^{-1}cm^{-1}. **Λ$_M$ (DMSO)** = 1.22 Scm^{2}mol^{-1}. **FTIR (KBr)** 3417, 3058, 1708, 1691, 1603, 1590, 1561, 1491, 1410, 1353, 1288, 1269, 1231, 1149, 1099, 854 cm^{-1}.

S2. Synthesis of 2-(2-oxo-2*H*-chromen-substituted-yl)oxyacetato copper(II)] complexes

The copper(II) complexes were synthesised by using a similar method as earlier described for the synthesis of zinc(II) complexes. The experimental data recorded for complexes **21-30** are given in the following paragraphs.

*S2.1. bis-{2-(2-Oxo-2H-chromen-3-yl)oxyacetatotetraaqua} copper(II) [Cu(C-3oxy-acet)$_2$.4H$_2$O] (**21**)*

Ligand = C-3oxy-acetH **(1)** (0.22 g, 1.0 mmol). **Yield** = 0.19 g, 69%. **M.P**. =230 °C (decomposed). **Solubility** = DMSO only. **CHN:** Calculated for $C_{22}H_{22}CuO_{14}$ %C, 46.04%, %H, 3.86%, %Cu, 11.07; Found %C, 45.97, %H, 3.63, %Cu, 11.17. **UV-Vis (DMSO)** λ = 765 nm ε = 92 M^{-1}cm^{-1}, λ = 325 nm ε = 19880 M^{-1}cm^{-1}, λ=310 nm ε=31960 M^{-1}cm^{-1}. **Λ$_M$ (DMSO)** = 1.77 Scm^{2}mol^{-1}. **FTIR (KBr)** 3477, 3065, 2926, 1715, 1626, 1575, 1450, 1403, 1381, 1280, 1164, 851 750 cm^{-1}.

S2.2. bis-{2-(2-Oxo-2H-chromen-4-yl)oxyacetatotetraaqua} copper(II)

*[Cu(C-4oxy-acet)$_2$.4H$_2$O] (**22**)*

Ligand = C-4oxy-acetH **(2)** (0.22 g, 1.0 mmol). **Yield** = 0.17 g, 63%. **M.P.** = 290 °C (decomposed). **Solubility** = DMSO only. **CHN:** Calculated for $C_{22}H_{22}CuO_{14}$ %C, 46.04%, %H, 3.86%, %Cu, 11.07; Found %C, 46.14, %H, 3.81, %Cu, 11.21. **UV-Vis (DMSO)** λ = 785 nm ε = 81 $M^{-1}cm^{-1}$, λ = 320 nm ε = 11540 $M^{-1}cm^{-1}$, λ = 305 nm ε = 21120 $M^{-1}cm^{-1}$, λ = 280 nm ε = 28900 $M^{-1}cm^{-1}$. **Λ_M (DMSO)** = 2.17 Scm^2mol^{-1}. **FTIR (KBr)** 3425, 3083, 2930, 1670, 1618, 1608, 1562, 1451, 1426, 1410,

1288, 1248, 839, 774 cm^{-1}.

S2.3. bis-{2-(6-Chloro-2-oxo-2H-chromen-4-yl)oxyacetatotetraaqua} copper(II)

*[Cu(6Cl-C-4oxy-acet)$_2$.4H$_2$O] (**23**)*

Ligand = 6Cl-C-4oxy-acetH **(3)** (0.25 g, 1.0 mmol). **Yield** = 0.20 g, 66%. **M.P.** = >300 °C (decomposed). **Solubility** = DMSO only. **CHN:** Calculated for $C_{22}H_{20}Cl_2CuO_{14}$ %C, 41.10%, %H, 3.14, %Cu, 9.89; Found %C, 41.14, %H, 3.27, %Cu, 10.03. **UV-Vis (DMSO)** λ = 785 nm ε = 75 $M^{-1}cm^{-1}$, λ = 325 nm ε = 13200 $M^{-1}cm^{-1}$, λ = 310 nm ε = 17880 $M^{-1}cm^{-1}$, λ = 280 nm ε = 25340 $M^{-1}cm^{-1}$. **Λ_M (DMSO)** = 2.38 Scm^2mol^{-1}. **FTIR (KBr)** 3421, 3102, 2989, 1709, 1627, 1566, 1432, 1410, 1371, 1288, 1230, 947, 824, 535 cm^{-1}.

S2.4. bis-{2-(4-Methyl-2-oxo-2H-chromen-6-yl)oxyacetatotetraaqua} copper(II)

*[Cu(4Me-C-6oxy-acet)$_2$.4H$_2$O] (**24**)*

Ligand = 4Me-C-6oxy-acetH **(4)** (0.23 g, 1.0 mmol). **Yield** = 0.18 g, 65%. **M.P.** = 260 °C (decomposed). **Solubility** = DMSO only. **CHN:** Calculated for $C_{24}H_{26}CuO_{14}$ %C, 47.88%, %H, 4.35%, %Cu, 10.56; Found %C, 47.54, %H, 4.17, %Cu, 10.37. **UV-Vis (DMSO)** λ = 750 nm ε = 126 $M^{-1}cm^{-1}$, λ = 340 nm ε = 11400 $M^{-1}cm^{-1}$. **Λ_M (DMSO)** = 1.88 Scm^2mol^{-1}. **FTIR (KBr)** 3429, 3083, 2973, 1720, 1646, 1572, 1494, 1428, 1383, 1276, 1171, 1062, 831 cm^{-1}.

S2.5. bis-{2-(2-Oxo-2H-chromen-7-yl)oxyacetatotetraaqua} copper(II)

*[Cu(C-7oxy-acet)$_2$.4H$_2$O] (**25**)*

Ligand = C-7oxy-acetH **(5)** (0.22 g, 1.0 mmol). **Yield** = 0.17 g, 65%. **M.P.** = 255 °C (decomposed). **Solubility** = DMSO only. **CHN:** Calculated for $C_{22}H_{22}CuO_{14}$ %C, 46.04%, %H, 3.86%, %Cu, 11.07; Found %C, 46.11, %H, 3.71, %Cu, 11.18. **UV-Vis (DMSO)** λ = 760 nm ε = 85 $M^{-1}cm^{-1}$, λ = 335 nm ε = 25980 $M^{-1}cm^{-1}$, λ = 325 nm ε = 31120 $M^{-1}cm^{-1}$. **Λ_M (DMSO)** = 1.67 Scm^2mol^{-1}. **FTIR (KBr)** 3410, 3090, 1730, 1686, 1604, 1561, 1420, 1402, 1283, 1232, 1144, 1042, 841 cm^{-1}

*S2.6. bis-{2-(4-Methyl-2-oxo-2H-chromen-7-yl)oxyacetatotetraaqua} copper(II) [Cu(4Me-C-7oxy-acet)$_2$.4H$_2$O] (**26**)*

Ligand = 4Me-C-7oxy-acetH **(6)** (0.23 g, 1.0 mmol). **Yield** = 0.19 g, 67%. **M.P**. = 290 °C (decomposed). **Solubility** = DMSO only. **CHN:** Calculated for $C_{24}H_{26}CuO_{14}$ %C, 47.88%, %H, 4.35%, %Cu, 10.56; Found %C, 47.92, %H, 4.20, %Cu, 10.80. **UV-Vis (DMSO)** λ = 755 nm ε = 124 $M^{-1}cm^{-1}$, λ = 335 nm ε = 28620 $M^{-1}cm^{-1}$, λ = 320 nm ε = 38660 $M^{-1}cm^{-1}$. **Λ_M (DMSO)** = 1.96 Scm^2mol^{-1}. **FTIR (KBr)** 3431, 3082, 2920, 1726, 1644, 1613, 1560, 1426, 1392, 1291, 1154, 1080, 848 cm^{-1}.

*S2.7. bis-{2-(3,4,8-Trimethyl-2-oxo-2H-chromen-7-yl)oxyacetatotetraaqua} copper(II) [Cu(3,4,8-triMe-C-7oxy-acet)$_2$.4H$_2$O] (**27**)*

Ligand = 3,4,8-triMe-C-7oxy-acetH **(7)** (0.26 g, 1.0 mmol). **Yield** = 0.19 g, 63%. **M.P**. = 270 °C (decomposed). **Solubility** = DMSO only. **CHN:** Calculated for $C_{28}H_{34}CuO_{14}$ %C, 51.10%, %H, 5.21%, %Cu, 9.66; Found %C, 51.10, %H, 5.26, %Cu, 9.41. **UV-Vis (DMSO)** λ = 750 nm ε = 87 $M^{-1}cm^{-1}$, λ = 320 nm ε = 25460 $M^{-1}cm^{-1}$. **Λ_M (DMSO)** = 2.07 Scm^2mol^{-1}. **FTIR (KBr)** 3422, 2927, 1710, 1642, 1605, 1576, 1499, 1426, 1382, 1347, 1285, 1131, 1078, 816, 763 cm^{-1}.

*S2.8. bis-{2-(3-Chloro-4-methyl-2-oxo-2H-chromen-7-yl)oxyacetatotetraaqua} copper(II) [Cu(3Cl-4Me-C-7oxy-acet)$_2$.4H$_2$O] (**28**)*

Ligand = 3Cl-4Me-C-7oxy-acetH **(8)** (0.27 g, 1.0 mmol). **Yield** = 0.19 g, 62%. **M.P**. = 285 °C (decomposed). **Solubility** = DMSO only. **CHN:** Calculated for $C_{24}H_{24}Cl_2CuO_{14}$ %C, 42.97%, %H, 3.61%, %Cu, 9.47; Found %C, 45.74, %H, 3.38, %Cu, 9.60. UV-Vis (DMSO) λ = 760 nm ε = 104 $M^{-1}cm^{-1}$, λ = 335 nm ε = 33780 $M^{-1}cm^{-1}$, λ = 325 nm ε = 35260 $M^{-1}cm^{-1}$. **Λ_M (DMSO)** = 2.97 Scm^2mol^{-1}. **FTIR (KBr)** 3430, 3082, 2923, 1735, 1617, 1570, 1508, 1424, 1382, 1342, 1288, 1204, 1156, 1082, 1008, 831, 753 cm^{-1}.

*S2.9. bis-{2-(4-(Trifluoromethyl)-2-oxo-2H-chromen-7-yl)oxyacetatotetraaqua} copper(II) [Cu(4CF$_3$-C-7oxy-acet)$_2$.4H$_2$O] (**29**)*

Ligand = 4CF$_3$-C-7oxy-acetH **(9)** (0.29 g, 1.0 mmol). **Yield** = 0.22 g, 67%. **M.P**. = 265 °C (decomposed). **Solubility** = DMSO only. **CHN:** Calculated for $C_{24}H_{20}CuF_6O_{14}$ %C, 40.70%, %H, 2.84%, %Cu, 8.95; Found %C, 40.70, %H, 2.89, %Cu, 8.80. **UV-Vis (DMSO)** λ = 765 nm ε = 98 $M^{-1}cm^{-1}$, λ = 340 nm ε = 30320 $M^{-1}cm^{-1}$, λ = 325 nm ε = 28320 $M^{-1}cm^{-1}$. **Λ_M (DMSO)** = 1.42 Scm^2mol^{-1}. **FTIR (KBr)** 3433, 3097, 2927, 1749, 1613, 1561, 1406, 1345, 1278, 1203, 1141, 1060, 875, 840, 731, 652 cm^{-1}.

*S2.10. bis-{2-(8-Acetyl-2-oxo-2H-chromen-7-yl)oxyacetatotetraaqua} copper(II) [Cu(8acetyl-C-7oxy-acet)$_2$.4H$_2$O] (**30**)*

Ligand = 8acetyl-C-7oxy-acetH **(10)** (0.26 g, 1.0 mmol). **Yield** = 0.20 g, 65%. **M.P**. = 255 °C

(decomposed). **Solubility** = DMSO only. **CHN:** Calculated for $C_{26}H_{26}CuO_{16}$ %C, 47.46%, %H, 3.98%, %Cu, 9.66; Found %C, 47.33, %H, 3.75, %Cu, 9.62. **UV-Vis (DMSO)** λ = 765 nm ε = 98 $M^{-1}cm^{-1}$, λ = 335 nm ε = 30100 $M^{-1}cm^{-1}$, λ = 325 nm ε = 37120 $M^{-1}cm^{-1}$. **Λ_M (DMSO)** = 1.21 Scm^2mol^{-1}. **FTIR (KBr)** 3483, 3084, 2998, 2925, 1732, 1720, 1692, 1625, 1603, 1565, 1407, 1351, 1273, 1150, 1107, 1096, 841 cm^{-1}.
